# 1736. Routine Vaccination Status at Time of Transplant for Pediatric Liver, Kidney and Heart Recipients

**DOI:** 10.1093/ofid/ofad500.1567

**Published:** 2023-11-27

**Authors:** Christopher Reis, Elizabeth H Ristagno, Theresa Madigan

**Affiliations:** Mayo Clinic, Rochester, Minnesota; Mayo Clinic, Rochester, Minnesota; Mayo Clinic, Rochester, Minnesota

## Abstract

**Background:**

Vaccine preventable infections (VPIs) are a significant cause of hospitalizations and morbidity and mortality for pediatric solid organ transplant (SOT) recipients. In this single-center study, we assessed up-to-date (UTD) status of routine childhood immunizations among our institution’s pediatric SOT recipients.

**Methods:**

We collected immunization data on all pediatric patients who underwent liver, kidney, or heart transplants between January 1, 2011 and December 31, 2021 at Mayo Clinic, Rochester, MN by retrospectively reviewing electronic medical record data. We also assessed whether patients were evaluated by a pediatric infectious diseases (PID) specialist within 1 year of transplant. Immunization status was determined using the CDC child and adolescent immunization schedule, excluding seasonal influenza and SARS-CoV-2 immunizations.

**Results:**

A total of 143 patients were included in our study (n = 45 liver, 54 kidney, 44 heart recipients); 49.7% were male. At the time of transplant, 39 patients were considered UTD (27.3% [Figure 1]). There was no difference by organ type (p = 0.59) and whether patients had seen PID prior to transplant (p = 0.17). Excluding live viral vaccinations, 45 patients were UTD (31.5%). Human papillomavirus was the least UTD vaccine series (Figure 2). Most patients were not UTD in just one or two series (Figure 1). A significant difference in UTD status was found based on age at transplant (p = 0.03), with younger patients having higher rates of UTD status (mean age 7.1 [SD 5.75] vs 9.9 [SD 5.92] years).

**Figure 1.**
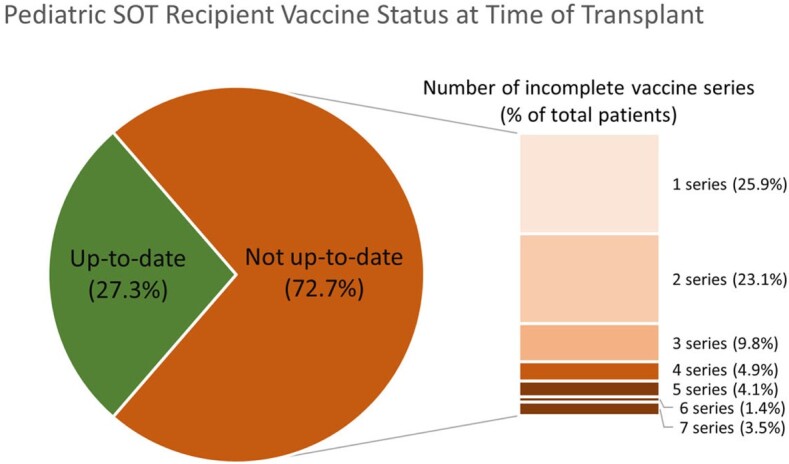
Vaccination status of pediatric SOT recipients at time of transplant and number of incomplete series.

**Figure 2.**
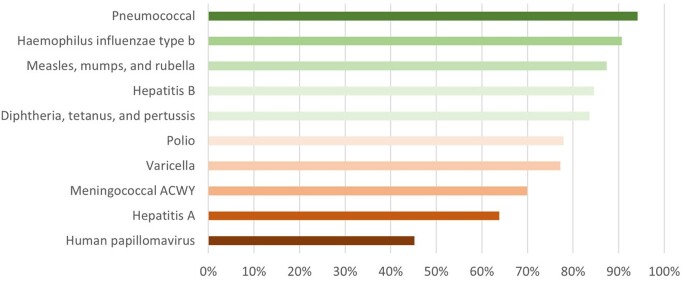
Vaccination status by series for eligible pediatric SOT recipients.

**Conclusion:**

To date, this study is the largest cohort of pediatric SOT recipients of three organ types assessing routine childhood vaccination status. In this single-center cohort spanning 11 years, we found that less than a third of patients were UTD on routine childhood immunizations at the time of transplant. While some immunizations can be given post-transplant, every effort should be made to update vaccines among transplant candidates to optimize efficacy and protection, thereby reducing the consequences of VPIs within this vulnerable population. Further studies are needed to address reasons for under-immunization and develop strategies to improve vaccination rates.

**Disclosures:**

**All Authors**: No reported disclosures

